# “The most culturally safe training I’ve ever had”: the co-design of a culturally safe Managing hepatitis B training course with and for the Aboriginal health workforce of the Northern Territory of Australia

**DOI:** 10.1186/s12913-023-09902-w

**Published:** 2023-08-31

**Authors:** Kelly Hosking, Teresa De Santis, Emily Vintour-Cesar, Phillip Merrdi Wilson, Linda Bunn, George Garambaka Gurruwiwi, Shiraline Wurrawilya, Sarah Mariyalawuy Bukulatjpi, Sandra Nelson, Cheryl Ross, Paula Binks, Phoebe Schroder, Joshua S. Davis, Sean Taylor, Christine Connors, Jane Davies, on behalf of the Hep B PAST partnership

**Affiliations:** 1 Northern Territory Health, Darwin, NT Australia; 2 Menzies School of Health Research, Charles Darwin University, Darwin, NT Australia; 3 Miwatj Aboriginal Health Corporation, Nhulunbuy, East Arnhem Land, Northern Territory, Australia; 4 Australasian Society for HIV, Viral Hepatitis and Sexual Health Medicine, Sydney, NSW Australia; 5 John Hunter Hospital, Newcastle, NSW Australia

**Keywords:** Aboriginal and Torres Strait Islander health, Aboriginal health workforce, Cultural safety, Health education, Training, Equity, Co-design, Hepatitis B

## Abstract

**Background:**

The Aboriginal health workforce provide responsive, culturally safe health care. We aimed to co-design a culturally safe course with and for the Aboriginal health workforce. We describe the factors which led to the successful co-design, delivery, and evaluation of the “Managing hepatitis B” course for the Aboriginal health workforce.

**Methods:**

A Participatory Action Research approach was used, involving ongoing consultation to iteratively co-design and then develop course content, materials, and evaluation tools. An Aboriginal and Torres Strait Islander research and teaching team received education in chronic hepatitis B and teaching methodologies. Pilot courses were held, in remote communities of the Northern Territory, using two-way learning and teach-back methods to further develop the course and assess acceptability and learnings. Data collection involved focus group discussions, in-class observations, reflective analysis, and use of co-designed and assessed evaluation tools.

**Results:**

Twenty-six participants attended the pilot courses. Aboriginal and Torres Strait Islander facilitators delivered a high proportion of the course. Evaluations demonstrated high course acceptability, cultural safety, and learnings. Key elements contributing to success and acceptability were acknowledging, respecting, and integrating cultural differences into education, delivering messaging and key concepts through an Aboriginal and Torres Strait Islander lens, using culturally appropriate approaches to learning including storytelling and visual teaching methodologies. Evaluation of culturally safe frameworks and findings from the co-design process led to the creation of a conceptual framework, underpinned by meeting people’s basic needs, and offering a safe and comfortable environment to enable productive learning with attention to the following: sustenance, financial security, cultural obligations, and gender and kinship relationships.

**Conclusions:**

Co-designed education for the Aboriginal health workforce must embed principles of cultural safety and meaningful community consultation to enable an increase in knowledge and empowerment. The findings of this research can be used to guide the design of future health education for First Nations health professionals and to other non-dominant cultures. The course model has been successfully transferred to other health issues in the Northern Territory.

**Supplementary Information:**

The online version contains supplementary material available at 10.1186/s12913-023-09902-w.

## Background

Globally, community health workers have been recognised as essential to enable comprehensive and effective primary health care [1]. Across Australia, the Aboriginal health workforce with their unique, diverse skill sets are crucial assets within the health system and play a vital role in contributing to the wellbeing of Aboriginal and Torres Strait Islander Peoples. The Aboriginal health workforce are most often located in primary health care services and view their role as strength-based, performing “*core functions of health promotion, clinical service and cultural brokerage*” [2]. Despite their professional value and contribution, it has been found that ongoing racially discriminatory structures and limited opportunities and benefits are pervasive for the Aboriginal health workforce [3]. In Australia’s Northern Territory (NT), health professionals, in general, are affected by high rates of staff turnover and burnout [4], including ongoing challenges to recruitment and retention for the Aboriginal health workforce [3]. It has been repeatedly highlighted that robust models that better support the Aboriginal health workforce [4, 5] are essential to improve chronic disease care [6] and reduce health inequities.

The NT covers a large geographic area, is sparsely populated and has the highest proportion of Aboriginal and Torres Strait Islander Peoples (26.3%, 61,000 people) in Australia, of which 76.6% reside in remote or very remote areas [7]. The NT has a rich and diverse culture, with over 100 Aboriginal and Torres Strait Islander languages and dialects spoken [8]. The NT has the lowest life expectancy in Australia [9], with a wide gap between Aboriginal and Torres Strait Islander and non-Indigenous Peoples, which is largely due to the effects of the social, cultural, and commercial determinants of health [10], stemming from colonisation and racism. Although the gap between Aboriginal and Torres Strait Islander and non-Indigenous peoples has recently narrowed by 26% for men (from 20.8 to 15.4 years) and 21% for women (from 19.5 to 15.4 years), it remains substantial [9] and is not on track to achieve the national target of “no gap” by 2031 [10]. The prevalence of chronic diseases is disproportionally high [10, 11], this unequal burden is reflected in chronic hepatitis B (CHB). NT Aboriginal and Torres Strait Islander Peoples have an estimated prevalence of 6.1% (ranging between 3–12%) [12, 13], compared to < 1% nationally [14]. If left unmonitored and untreated CHB will lead to liver cancer and cirrhosis in 25% of people [15, 16]. CHB is a lifelong condition, as such, the primary health care workforce, including the Aboriginal health workforce, is well placed to provide high-quality care for people living with CHB. However, research has found that CHB is often misunderstood by those living with the condition, their families, and communities, and those involved in their care and management [17–19]. CHB-specific education exists for doctors and nurses but not for the Aboriginal health workforce. We aimed to address this gap in CHB education and increase equitable access to educational opportunities by co-designing, developing, and delivering a culturally safe training course for the Aboriginal health workforce.

### Cultural safety

The terms cultural competency and cultural safety are often interchanged. However, differentiation between the terms is important. Cultural competency is the achievement of measurable skills, knowledge, and attitudes, which theoretically leads one to become aware of their own culture whilst providing care to diverse populations [20]. Cultural safety was originally described by Māori nurse Dr Irihapeti Ramsden who explained that we live in a neo-colonial environment that requires a profound understanding of the social function of racism and the colonial process [21]. Cultural safety extends beyond competency, with an emphasis of dignity and respect and is only determined by the recipient of the exchange, in our context Aboriginal and Torres Strait Islander individuals, families and communities [22, 23]. An abundance of frameworks exist describing cultural safety and cultural safety training for health professionals to guide culturally safe health care (for the clinician patient relationship) [22–28]. While it is recognised as essential that Aboriginal and Torres Strait Islander Peoples have access to meaningful training options [29], less is described with respect to creating culturally safe education for the Aboriginal health workforce. Distinct from cultural safety training for health professionals, this paper documents a process of developing a culturally safe training course for the Aboriginal health workforce.

## Methods

### Researcher reflexivity

Most of the team have worked together for many years and have built trusted relationships through shared underlying values. Although strong hierarchies can exist in health, our team has deep respect for each role, with none being more important to patient outcomes than the other. The Aboriginal and Torres Strait Islander members of the team come from diverse language and cultural groups throughout the NT and Torres Strait Islands. TDS, a Tiwi woman, has been an Aboriginal Health Practitioner (AHP) for 19 years. She had family or kinship relationships with the participants in pilot 2. PMW, AHP and artist, is a Ngangi speaker and lives in Nauiyu community, on Malak Malak land. SW, a Warnidilyakwa woman and Anindilyakwa speaker from Groote Eylandt, has worked as an AHP for over 20 years. SN, a Gurindji woman and AHP for 24 years. GG is an Aboriginal Community Worker (ACW), researcher and proud Yolŋu man. SB is a Yolŋu woman and senior AHP. CR is a proud Arrernte, Kaytete woman and has extensive family and kinship relationships across the NT. LB, an AHP for over 40 years, grew up in West Arnhem Land with Iwaidja speaking and mainland Kunwinku clans. ST is a descendent of the Dauareb Tribe, of Mer (Murray) Island in the Torres Strait with over 20 years clinical and research experience. The non-Indigenous members of the team include nurses and PhD candidates (KH, PB) and doctors (JD, JSD, CC) and hepatitis program managers (EVC, PS) with a combined experience in Aboriginal and Torres Strait Islander health of over one hundred years. As decolonisation researchers we are critically conscious of the impacts and damages inflicted by colonisation [22]. We acknowledge our position of privilege and reflect on and act to remove any potential biases [22, 30, 31]. We are grateful to live and work on Aboriginal and Torres Strait Islander land and understand that we are on a continuing learning journey.

### Study design and process

This study is part of a larger Participatory Action Research (PAR) project, Hep B PAST, a partnership approach to sustainability eliminate CHB from the Aboriginal and Torres Strait Islander population of the NT [32]. The project aims to improve CHB-related health literacy, clinical care and the cascade of care for people living with CHB [32]. A key component to support primary health care to manage CHB is capacity building of health professionals through the delivery of CHB education for doctors, nurses, and the Aboriginal health workforce. As there was no specific CHB-related education for the Aboriginal health workforce we aimed to fill this gap, through the co-design and development of the “Managing hepatitis B” course.

### Theory of co-design

**Fig. 1:**
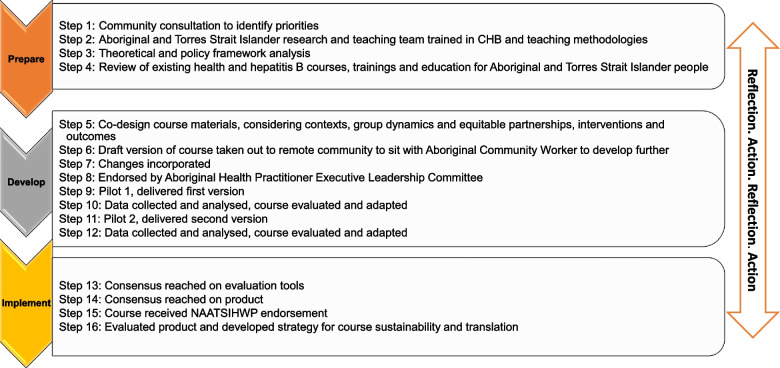
The process for the co-design of our education course for the Aboriginal health workforce

Co-design is emerging as an empowering methodology for Aboriginal and Torres Strait Islander Peoples and is a culturally grounded, respectful approach that involves true partnerships [33]. Underpinning co-design methodology for health programs and research are principles of collaboration, empowerment, and capacity building. To achieve these principles, research must be flexible, undertaken with respect, enact shared decision making and have equitable accountability [33]. Achieving effective co-design takes time and researchers must allow ethical space, which is needed when peoples from disparate worlds and cultures engage in work together [34]. The process for the co-design of the course is summarised in Fig. 1.

### Prepare

#### Step 1: Community consultation to identify priorities.

The development of this course used an iterative, PAR approach involving extensive consultation with the Aboriginal health workforce to ensure the content was appropriate and reflected contexts of community. PAR reflection acknowledges difference, history and trauma and is a decolonising and empowering methodology [35]. This step continues throughout the entire process.

#### Step 2: Aboriginal and Torres Strait Islander research and teaching team trained in CHB and teaching methodologies.

The research and teaching team consisted of AHP coordinators (TDS, SW, SN, LB), who provide support to the Aboriginal health workforce. Although skilled in primary health care and in supporting the workforce, they had no previous CHB training. Other team members brought skills in chronic conditions, CHB management and research (GG, PMW, SB, CR, ST). SB and GG were instrumental in the development of the Hep B story app [36] and the “one stop liver shop” a holistic mobile care model to deliver CHB care in community [37]. The team were provided training and mentoring and participated in multiple workshops (led by KH) to increase their knowledge and skills about CHB management and teaching methodologies*.*

#### Step 3: Theoretical and policy framework analysis

Adult learning principles and resources [38] and Aboriginal and Torres Strait Islander pedagogy frameworks [39, 40] were considered. The theoretical framework and reflective processes of this study are informed by cultural safety frameworks [21, 22, 24, 28], social ecological models [41] and Freirean pedagogy [30], understanding that participants are co-creators of knowledge and recognising there must be action with reflection and reflection with action [30]. Practicing pedagogy that includes critical consciousness allows an awareness of social, economic and political systems that oppress people [42], and involves honestly confronting realities, listening to participants lived experience and solutions and critically reflecting how to change a system of oppression [30]. A social ecology model modifies education within the social and ecological landscape [41] to assist knowledge translation into practices and transmission of accurate and culturally relevant health messages. Policies and frameworks that consider First Nation’s human rights and cultural safety were reviewed and considered in the development of this course, of which eleven are summarised in Table 1. The interface of these theories, policies, frameworks, and health science were enhanced with the insights and methods from Aboriginal and Torres Strait Islander knowledge and belief systems and the valued input from our Aboriginal and Torres Strait Islander research and teaching team and participants [43].

**Table 1:** Policy Framework review

**Document**	**Source**	**Goal **	**Key points and principles **
NT Health Aboriginal Cultural Security Framework 2016-2026	[23]	Accessible and effective health care systems for Aboriginal people based on the right of Aboriginal self-determination and access to health care.	Key points: Improve retention, provide support and training to achieve career and life goals. Consider ways to build, strengthen and reward local workforce in remote areas. Training to increase awareness of Aboriginal cultures. Values skilled and culturally reflective workforce and has a focus to develop the Aboriginal workforce.
NT Aboriginal Health Plan 2021-2031	[44]	Includes goal to strengthen the health workforce	Key points: Action, reflection, action, learning. Key principles; cultural respect, community control, ethical practice, health equity and accessibility
National Agreement on Closing the Gap	[10]	To overcome inequality faced by Aboriginal and Torres Strait Islander Peoples so that life outcomes are equal.	Key points: Shared decision making; building community-controlled sector; transforming government organisations, Aboriginal and Torres Strait Islander-led data
National Health and Medical Research Council. Ethical conduct in research with Aboriginal and Torres Strait Islander Peoples and communities: Guidelines for researchers and stakeholders	[45]	To provide a set of principles to ensure research is safe, respectful, responsible, high quality, and of benefit to Aboriginal and Torres Strait Islander Peoples and communities	Key principles: cultural continuity, equity, reciprocity, respect, responsibility and spirit and integrity
National Aboriginal and Torres Strait Islander Health Workforce Strategic Framework and Implementation Plan 2021–2031	[27]	Increase Aboriginal and Torres Strait Islander Health Workforce. Strengthen the health system including improving the attraction, retention, and career development of Aboriginal health staff	Key points: access to continuity of education, racism causing a crippling impact on education, workforce recruitment and retention. Needs to improve and strengthen cultural safety within education and training and across health workforce. Key principles: centrality of culture, leadership and accountability, partnership, health system effectiveness and evidence of data.
NATSIHWA Cultural Safety Framework National Aboriginal and Torres Strait Islander Health Workers Association	[24]	Increase the capacity within the healthcare system to deliver culturally safe and responsive health and well-being services for Aboriginal and Torres Strait Islander Peoples.	Key points: Critical to increase the understanding of the role and value of Aboriginal and Torres Strait Islander Health Workers across the health system. Key principles: Aboriginal self-determination, social and restorative justice, equity, negotiated partnership, transparency, reciprocity, accountability, sustainability, political bipartisanship, cultural contextuality
(AHPRA) National Scheme's Aboriginal and Torres Strait Islander Health and Cultural Safety Strategy 2020–2025	[22]	Eliminate racism from the health system and to have a culturally safe health workforce through nationally consistent standards, codes, and guidelines across all practitioner groups.	Key points: To ensure culturally safe and respectful practice one must acknowledge colonisation, systemic racism, social, cultural, behavioural and economic factors which impact health; address individual racism, their own biases, assumptions, stereotypes and prejudices, recognise the importance of self-determined decision-making, partnership and collaboration, foster a safe working environment through leadership to support the rights and dignity of Aboriginal and Torres Strait Islander Peoples and colleagues
National Aboriginal and Torres Strait Islander Health Plan 2013-2023	[26]	Aims to have a health system free of racism and inequality and all Aboriginal and Torres Strait Islander Peoples have access to health services that are effective, high quality, appropriate and affordable. Implement cultural safety and quality of care across the entire health system.	Key points: recruitment of Aboriginal and Torres Strait Islander Peoples in the health workforce, retention in rural and remote and culturally competent workforce. Development of health workforce. Key principles: Health equality and a human rights approach; community control; partnership; accountability.
National Aboriginal Community Control Health Organisation, Creating the NACCHO Cultural Safety Standards and Assessment Process	[28]	Creating an environment of cultural safety in health services to ensure responsive and culturally appropriate care	Key points: Cultural differences are respected. Including the right to achieve equitable health outcomes. The Framework emphasises knowledge and awareness, skilled practice and behaviour, strong relationships between Aboriginal people and communities, and the health agencies providing services to them, including Aboriginal staff.
Cultural safety in health care for Indigenous Australians: monitoring framework	[25]	Health system that respects Indigenous cultural values, strengths, and differences, and addresses racism and inequity	Key point: The Indigenous workforce is integral to ensuring that the health system addresses the health needs of Indigenous Australians in a culturally safe and sensitive way
United Nations Declaration on the Rights of Indigenous Peoples	[46]	The Declaration is a comprehensive statement addressing the human rights of Indigenous Peoples to live in dignity, to maintain and strengthen their own cultures and traditions and to pursue their self-determined development.	Key principles: justice, democracy, respect for human rights, non-discrimination, and good faith.

#### Step 4: Review of existing health and hepatitis B courses, trainings, and education for Aboriginal and Torres Strait Islander Peoples.

We reviewed literature on courses, training, and education for the Aboriginal health workforce [47–53] as well as health and health literacy education for Aboriginal and Torres Strait Islander communities [54, 55]. There were no existing CHB courses specifically designed for the Aboriginal health workforce, nor First Nations peoples globally.

### Develop

#### Step 5: Co-design course materials, considering contexts, group dynamics and equitable partnerships, interventions, and outcomes.

Authors KH, JD, PB are involved in the development and delivery of the national hepatitis B courses for doctors and nurses, with the peak organisation for the sector, Australasian Society for HIV, Viral Hepatitis and Sexual Health Medicine (ASHM). The structure of the existing ASHM course and some graphics were used as a basis to commence design. We reviewed and utilised some content from St Vincent’s hospital’s education tool in plain English “the hepatitis B story” [56]. Workshops were conducted with the Aboriginal and Torres Strait Islander research and teaching team to develop and refine course content, training materials, role plays, case studies, games, and evaluation tools. Materials were adapted considering context, group dynamics and equitable partnerships, interventions, and outcomes [57]. As the course was aimed at the wider Aboriginal health workforce (rather than only the more clinical AHP profession) the course content was developed to accommodate varying literacy and professional levels with the flexibility to break into smaller discrete subject groups. The course topics included the role of the Aboriginal health workforce in CHB care, epidemiology, transmission, prevention, anatomy and physiology of the liver, health promotion and liver health, CHB management, care and support, testing, serological interpretation, CHB phases and treatment and how to use the Hep B story [36]. At key points throughout the process, we sat together to assess what each slide and concept meant and considered if images were culturally appropriate iteratively including alternative methods or stories to improve the teaching of key concepts.

#### Step 6: Draft version of course taken out to remote community to sit with Aboriginal Community Worker to develop further.

We (KH, JD) took the first iteration of the course to a very remote community for consultation with senior Aboriginal Community Worker (ACW). GG is a traditional man, whose first language is Yolŋu Matha. He offered an important perspective as the only male on our team initially and as an ACW, a non-clinical role, to ensure content was relevant, clear, and appropriate.

#### Step 7: Changes incorporated

Changes were made to the course materials based on the feedback, with the updated version reviewed to ensure we captured and articulated requirements and concepts correctly.

#### Step 8: Endorsed by Aboriginal Health Practitioner Executive Leadership Committee

The course material was presented for review and endorsement by the Top End Health Service AHP Executive Leadership Committee, a professional advisory committee that provides clinical governance, leadership and is a decision-making authority regarding AHP practice and professional matters.

#### Step 9 and 11 –Pilot 1 & 2 – delivery of course

The course was piloted in two remote communities in 2019. These communities were purposively chosen as authors (KH, LB, JD, CC) had existing working relationships with the community and health staff.

#### Step 10 and 12: Data collected and analysed, course evaluated and adapted.

Participants provided expertise in cultural context, stories, and acceptability. Facilitators collected data through evaluation tools, in-class observations, small group discussions and through reflective journaling. The teach-back method [58] and knowledge assessment tool, based on the core learning outcomes of the course (see Additional file 1) were used by facilitators during case studies and role plays to assess competence and highlight areas where content need to be clearer.

### Implement

#### Step 13: Consensus reached on evaluation tool

Focus group discussions were conducted with the Aboriginal and Torres Strait Islander research and teaching team to trial and develop evaluation tools. Tools were further tested, through in-class observation, focus groups and voting to ascertain which evaluation methods were acceptable to the participants (see Additional file 1).

#### Step 14: Consensus reached on product

We agreed upon a base version of the course and materials, understanding that iterative improvement may still be required, including incorporating new evidence and adaptions for different communities.

#### Step 15: Course received National Association of Aboriginal and Torres Strait Islander Health Workers and Practitioners (NAATSIHWP, formerly NATSIHWA) endorsement

NAATSIHWP is the peak workforce association for Aboriginal and Torres Strait Islander Health Workers and Practitioners. Endorsement was provided after demonstrating that the course met NAATSIHWP's five overarching standards of respect and culture; purpose and knowledge; skills and competence; active learning and relevance.

#### Step 16: Evaluated product and developed strategy for course sustainability and translation

PAR principles were used to write this paper to capture and reflect the Aboriginal health workforce voice. Two full-day workshops with four of our Aboriginal and Torres Strait Islander research and teaching team attending. A draft paper was distributed prior to each workshop. Terminology and definitions were agreed, and each section was analysed and reworked to ensure consensus. Care was taken to portray our participants’ stories accurately and respectfully.

## Results

### Prepare

The review of eleven policies and frameworks around cultural safety and Aboriginal and Torres Strait Islander health and education revealed strong themes of addressing racism and the need to improve equity in healthcare and health outcomes for Aboriginal and Torres Strait Islander Peoples [10, 22–24, 26, 44–46]. The value of the Aboriginal health workforce was highlighted, as was the need to improve recruitment and retention and strengthen the Aboriginal health workforce, including the need for greater education and training opportunities [23–27, 44]. There was no clear implementation plan or framework on how to deliver culturally safe training for the capacity building of the Aboriginal health workforce. We considered all key principles, with NAATSIHWP and NHMRC frameworks most closely aligned to what we believe essential to achieve culturally safe training. With the foundation principle of spirit and integrity, we integrated the principles of reciprocity; respect; cultural contextuality and continuity; equity and access; responsibility and advocacy and sustainability [24, 45] with the overarching theory of the empowering potential grounded in cultural safety and decolonisation [21, 30, 57]. Whilst not explicitly mentioned in the frameworks reviewed, a strong and consistent finding throughout the iterative process of course development was that values described in Maslow’s Hierarchy of human needs [59] are critical for participants to be empowered and safe in the learning environment and to maximise critical thinking and learning potential. This involves a holistic approach, including physiological, safety, love and belonging, and self-actualisation [59].

**Fig. 2:**
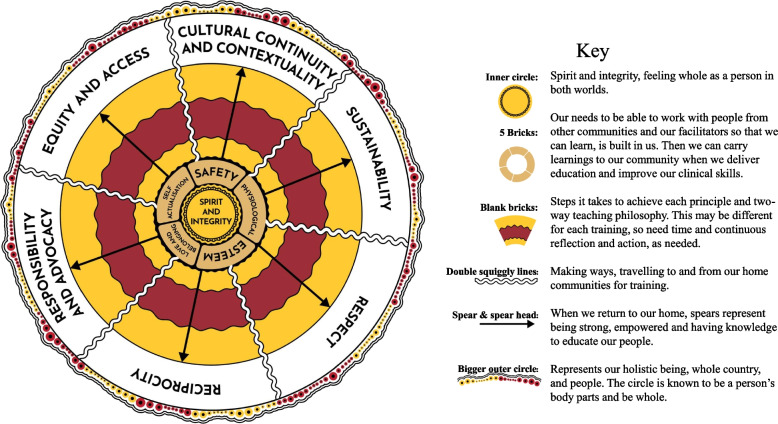
Process model to develop culturally safe training, adapted from cultural safety frameworks [24, 45] and Maslow’s hierarchy [59]

A conceptual process model for the co-design and development of culturally safe education for Aboriginal and Torres Strait Islander Peoples was conceived. This was further refined through the writing process with the Aboriginal and Torres Strait Islander research and teaching team, with author PMW putting the concepts into art and story, depicted in Fig. 2. We understand that this study has been co-designed as part of research and that health educators may be more time limited. The conceptual process model in Fig. 2 can be utilised considering the principles and values described in the outer and inner circles and filling in the ochre and brown sections with the steps taken to achieve each principle. Table 2 describes practical examples of how to demonstrate alignment with principles, as a guide.

**Table 2:** Alignment of course design with cultural safety principles, adapted from NAATSIHWP [24] & NHMRC [45]

**Cultural safety principle**	**Demonstration of alignment**	**Evidence and practical examples**	**Self-reflection achievement met/not met**
**Reciprocity** Shared responsibility and obligation based on kinship networks. Shared mutual benefit.	Equitable and respectful engagement Ensuring Aboriginal and Torres Strait Islander Peoples and communities have the right to define benefits according to their own values and priorities. Linked to local, national health priorities.	Two-way learning providing a safe space for participants to discuss content, facilitated by male and female trained Aboriginal health staff. Confirming the appropriateness of course content prior to delivery with local staff based in community. Content of course modified in accordance with participant feedback and community needs e.g. *“This course should be longer”,* we changed to 1.5 days. *“We should come together again and talk about hep b”* we organised in-services, visited community and invited participants back to subsequent course. We enhance community capacity beyond the project, including transferable skills in chronic conditions management and transferrable model of education. Borne from community consultation for a need to improve CHB care on country and training for Aboriginal health workforce. The project was linked to community and national health priorities and strategies.	Met
**Respect**	Respected decision processes of community Minimised difference blindness and engage with Aboriginal and Torres Strait Islander knowledge and experience. Respected cultural obligations and commitments. Respecting difference in education levels, professional expertise, and catering to all levels	Developing respectful relationships Use of metaphors and allowed time for and storytelling. Giving opportunity for facilitators and participants. Flexible and responsive, centring cultural priorities*. “I felt very safe”.* Course tailored to different education and literacy levels. Participants could break into groups of their choice. Verbal and storytelling used, and facilitators explained – not relying heavily on written text or answers	Met
**Equity and access** Showing fairness and justice Benefits from research show flow mostly to the community, not the researchers. Integrating Aboriginal and Torres strait Islander worldviews into programs is critical to achieve culturally safe transformational change. Seeks to identify and redress historical, social, and political imbalances and inequities	Equitable partnerships Ensuring equality and equity to educational opportunities Actively engaging community in to and methods & considering first language and communication	Co-designed course. Ensuring equality and equity to educational opportunities. Delivering in remote/regional locations. Offered to broad range of Aboriginal and Torres Strait Islander workforce and ensuring course tailored to many educational and professional levels, respecting each other. Consciously considering factors including location, travel, accommodation, catering, and health clinic staffing, to remove barriers for attendance. Advocating for participant attendance by discussing the course with key stakeholders including Aboriginal health practitioner coordinators, district managers, clinic managers and other community health staff including doctors and nurses. Designed course content to suit a variety of learning styles, with a preference for visual, interactive, and kinaesthetic methods for a variety of educational and professional levels. Ability to make games, evaluation, knowledge sharing inclusive, so everyone has equal opportunity to contribute.	Met
**Cultural contextuality & ** **cultural continuity** A sense of strong, shared, and enduring individual and collective identities	Negotiated participation and awareness of cultural events (e.g., sacred sites; women’s business and men’s business). Establishing mechanisms that incorporate the balance between collective and individual identity. Establishing a community advisory group and respecting the community’s decisions. Considering the use of Aboriginal and Torres Strait Islander standpoints and methodologies when development	Acknowledging upfront that some concepts include culturally sensitive information. Before and during the course, acknowledge that some content (about transmission) will be talking about men’s and women’s business. Seeking permission and allowing participants to leave. Understanding kinship and ensuring gender balance of facilitators and participants. Working with community before selecting a training date to avoid dates of cultural significance. Understanding that the community are the experts in their culture and not being difference blind using strengths-based approaches. PAR and two-way approach. Aboriginal and Torres Strait Islander research team and participants informed development of the course. Reflecting, learning changing as required. *“This is the most culturally safe training I’ve ever had”.*	Met
**Responsibility and advocacy ** Caring for country, kinship, bonds, maintenance of cultural and spiritual awareness. To do no harm, including avoiding having an adverse impact on the ability of others to comply with their responsibilities	Treating people with respect as adult learners Responsibility to ensure basic needs are met (accommodation, food etc) Responsibility to support ongoing learning and development - ongoing follow up of participants, mentorship. Responsibility to ensure quality training materials to pass on knowledge.	We understood that people have other family and cultural responsibilities and allowed for this. Enabling and facilitated opportunities to do other things and meet obligations in “town”. Sharing responsibility of imparting knowledge to assist in critical thinking. Advocated with managers for travel allowance to be paid upfront to avoid financal stress. All food and accommodation provided. Provided on-going training opportunities, including assisting liver clinics, and provided mentors and support (i.e., AHP coordinator, nurse in clinic, where possible) Provided with education booklet, Hep B Story app, presentation with key messages. Manager informed before and after of learning outcomes and provided with information of how to support staff to consolidate learnings.	Met
**Sustainability** Sustained commitment to improving healthcare services to Aboriginal and Torres Strait Islander Peoples and educational opportunities for the Aboriginal health workforce.	Sustain partnerships and relationships with Aboriginal and Torres Strait Islander Peoples and communities that are based on trust, mutual responsibility, and ethics. Sustain education opportunity. Initiating research translation strategy	Developing trusted relationships through the design and delivery of the course, researchers and community developed relationships which has vast beneficial implications beyond the delivery of this course. *“Building relationships is crucial particularly in the NT where most clinics are in remote settings”.* Endorsement from NAATSIHWP. Presented findings to organisations and community. Successful advocacy and investment from NTG to fund future courses. Working with managers to support attendance in trainings. Initiating research translation strategy, using model for other health conditions. Successfully transferred model to COVID-19 training. Broader model of care, Aboriginal health workforce part of the core clinical care group. Improvements in the cascade of care for people living with CHB.	Met

### Develop

A full-day course was delivered in two communities. Seven participants, all ACWs, attended pilot one, which allowed us to assess if course materials were at the appropriate level for this group. Nineteen AHP students, from across Australia attended pilot 2. Ideally education should be delivered in someone’s preferred language, however the challenges with achieving this are exemplified in our first pilot where there were four distinct first languages (Burarra, Kunwinjku, Ndjebbana, Yolŋu Matha) amongst our seven participants. The pilot courses were delivered in English (by KH) using plain language. Aboriginal and Torres Strait Islander teaching team members (LB, TDS, SN) were seated at tables with participants to clarify messages in small groups.

Two-way (also called both-way) teaching philosophy was used, which positions Aboriginal and Torres Strait Islander Peoples as knowledge holders in all educational transactions and preferences, bringing together Western academic discipline, respectfully embracing diversity in all cultural contexts [60, 61]. It involves a continuing reflection of teaching and learning [60]. The course involved a mix of slides, activities, role plays, case studies and games. Case studies were developed to reflect the roles of the Aboriginal health workforce in CHB management in a remote NT context. Parallels and examples were drawn to other chronic conditions to increase utility and transferability of skills learnt and to assist in making links to a chronic conditions model of care. The course utilised the Hep B Story app, developed by our research group to improve health literacy about CHB by providing education in a person’s first language [36], currently available in nine NT Aboriginal and Torres Strait Islander languages [32].

### Pilot 1 Reflections

Reflective analysis and journaling (by KH, LB) occurred directly after the delivery of pilot one. Reflections included that overall, the participants were positive, actively engaged and were comfortable to ask questions and share stories. Observations and feedback throughout the course identified that although having training in community enabled more ACWs to attend it also led to distractions for the participants, who were not always able to fully engage due to being called out of class and having the *“little faces (of local children) looking through the windows.”* Group discussions with the participants confirmed these observations, with one participant saying. *“There’s too much humbug [harassment] if we stay (in community), its better if we go to Darwin [to learn].”*

Despite distractions, there were some clear learnings, including that hepatitis B was an ancient virus which has been present as an infection in Aboriginal and Torres Strait Islander Peoples for over 50,000 years [62]. This piece of knowledge was mentioned as important in group discussions and on butcher paper evaluation. One participant reflected. *“It [HBV] is not a “white fellas” disease, it is old, from our ancestor’s time.”*

All the participants expressed that the course was acceptable, and they enjoyed learning about CHB. They articulated they enjoyed the interactive and visual aspects of the course. With one participant commenting. *“It had pictures, some people are visual learners, so I found this helpful.”* and another, *“I really liked the fun games. The game about how you get hep b was good for me.”*

### Pilot 2 reflections

Reflective analysis and discussion after the course between the facilitators (KH, TDS, LB, and SN) found that there was excellent energy in the room and thorough engagement from the participants. Facilitators commented that throughout breaks the participants continued to ask questions about CHB and provided positive feedback on delivery methodologies, content and a feeling of cultural safety and respect.

In response to “the best thing about the course is,” one participant commented that it was, *“Culturally appropriate and safe”.* This sentiment was confirmed in group discussions, the feedback was overwhelmingly positive about course acceptability, with one participant reflecting. *“This was the most culturally safe training I’ve ever had.”*. The delivery and style of the course was described as *“fun”, “interesting”* and *“understandable,”*. The facilitators received positive reviews, with three mentions that *“The course was very well presented”* and *“the facilitators were deadly [awesome].”*

The acceptance of course delivery was supported through feedback in small group discussions, with participants again using terms *“understandable”* and *“fun.”* One participant commented*, “I love how it was so interactive.”.* To the question “If I could change the course, I would prefer….” The responses included three mentions of *“nothing,”* “*more games and videos*” and “*longer course.”* Through group discussions several people agreed that the course could be longer (than one full day) to consolidate learnings and further explore hepatitis B serological interpretation, which people found challenging.

### Cultural safety principles

The processes of course design was reviewed against culturally safe principles, adapted from NAATSIHWP and NHMRC [24, 45], used as process indicators. We measured evidence from the co-design process and the delivery of the pilot courses, describing practical examples. The research team reflected on whether each domain was achieved. We found that that course design closely aligned with culturally safe principles, see Table 2.

### Implement

The process of co-designing the “Managing hepatitis B” course began in 2018, with a 6-month evaluation to be ready for piloting. Two pilot courses were run, in remote communities in the NT, in 2019, with a total of twenty-six participants attending. The final product is a 1.5-day course which has been delivered eight times to over 150 participants, throughout the NT, between 2019 and 2023 [63]. Course materials and guidelines for use are available on request to corresponding author.

## Discussion

This study found that the process of co-design, and delivery of the “Managing hepatitis B” course was successful and culturally safe. Despite acknowledgement of the need to increase supply and retention and improve educational opportunities [23–27, 53] a gap was identified in describing how to implement culturally safe training for the Aboriginal health workforce. Overarching philosophies and pedagogy review identified themes relevant to our context [39, 40]. We drew from decolonising, social ecological and critical race theories, using reflective analysis to integrate concepts of cultural safety, inclusion, and power in relation to health and education [21, 30, 64]. The findings provided a valuable starting point to commence and shape the co-design of the “Managing hepatitis B” course. Through combining the learnings of our process of co-design with the theories and frameworks reviewed we were able to develop a process model for the development of culturally safe training.

### Alignment with cultural safety principles

Studies have shown that Aboriginal and Torres Strait Islander Peoples want and benefit from having an Aboriginal and Torres Strait Islander person involved in their health care [65–68]. Research in the NT has shown the AHP profession has seen a decline in numbers in the past decade [4]. Cultural safety, teamwork, collaboration, recognition, and professional development have been identified as crucial to the retention of Aboriginal health staff [4]. Our education was designed to increase capacity of the broader Aboriginal health workforce. Education should seek to build upon the skills and knowledge of the whole workforce and encourage the development of non-clinical skills, such as leadership or research [69], providing safe workplace environments, supervision, mentoring and offering opportunities for ongoing collaboration [48, 70]. This was embedded within our training, with our model encouraging past participants to become future facilitators, leading to numerous occasions where they were invited to share their knowledge in national and international settings [71–73]. Education should be guided by the principles of cultural safety and PAR in its program design and interactions. Participatory action research engenders change through both research outcomes and through the research process itself [74]. As well as contributing to course content and delivery, the Aboriginal and Torres Strait Islander research and teaching team developed Hep B games as a low-cost vehicle for education and helped shape the role plays and case studies to be relevant for various regions and cultural contexts in the NT. Being involved in the research process provided ongoing opportunities for two-way learning within the team and empowered participants to have an impact on how CHB care was delivered in their communities. Culturally respectful communication is another key component to delivering culturally safe health care [21, 55, 65]. This should extend to health education and include a focus of strengthening interprofessional relations and involve meaningful co-creation of knowledge, as outlined in NHMRC’s principles, specifically reciprocity [45]. The collaborative, two-way learning approach intrinsic to our education model, benefited both researchers and participants and led to an enriching, respectful and enjoyable learning experience which extended beyond the training. Our findings demonstrated alignment with the NAATSIHWP framework being informed by academic, clinical expertise and Aboriginal and Torres Strait Islander cultural knowledge [24]. We were acutely aware of the importance and applicability of both verbal and non-verbal Aboriginal and Torres Strait Islander communication styles and recognition and respect for cultural obligations and cultural validation. We acknowledged and respected cultural protocols, kinship, and gender specific issues. This is particularly important when teaching HBV transmission, which includes maternal to child transmission, sexual transmission, and blood-related transmission through ceremony. At the beginning of the course, we highlighted that we would need to discuss issues related to “men’s” and “women’s” business and gave people opportunities to voice concerns with facilitators. Aligned with the social ecological model our participants and facilitators described a sense of ownership over the course developing practical strategies for learning opportunities and behavioural change [41].

Like culturally safe health care for patients, culturally safe education should be developed through reflective practice based on experience and knowledge; reflection on one’s own cultural identity and recognition of the impact culture has on one’s own behaviour and practice. Culturally safe education should flatten the hierarchy between participants and educators to facilitate two-way principles of cross-cultural learning and empowerment. Cultural safety can only be defined by those people who receive services [24]. In our study we did not ask the question “is our training culturally safe?” this was independently determined and expressed by participants. Achievement of cultural safety was further defined by the Aboriginal and Torres Strait Islander research and teaching team through reflective practice, reviewing evidence from practical examples of our model, which demonstrated alignment to cultural safety principles [75].

### Flexibility of course co-design, content, and delivery

In their recent comprehensive review of co-design approaches with First Nations Peoples, Butler et al. found themes which would lead to success and which we applied to our co-design, including Aboriginal and Torres Strait Islander leadership, a culturally grounded approach, inclusive partnerships and evidence-based decision making [33]. We found the process of co-design is just as important as the product but to do it properly takes time and space to develop relationships and partnerships.

Flexible approaches to health education accommodate the various commitments, learning styles and skills specific for the Aboriginal health workforce and increase visual representations and interactive approach [51, 52]. These approaches allow the focus to be on the development of skills rather than traditional assessment focused, repetition-based training [76]. Literature suggests that Aboriginal and Torres Strait Islander Peoples learn more effectively when teaching includes kinaesthetic, contextual, role plays, practical session, observing and sharing strategies [48, 53, 77, 78]. Visual and interactive materials, games and stories telling, and case studies were all an integral part of the course. Aboriginal and Torres Strait Islander cultures have a strong oral history and allowing time for storytelling contributed positively to our course [78, 79]. Participants were given the opportunity to apply knowledge in contexts relevant to their everyday lives and given space to safely discuss community issues, sharing stories and challenges and discussing solutions with peers, fostering a collaborative, strengths-based approach to learning [80]. To improve sustainability of education and to encourage self-sufficiency post course, appropriate resources, key messages and ongoing mentoring and support was provided to participants. This is crucial is maintaining and building knowledge [48, 81, 82]. Similar to findings in other studies our participants requested refresher training and access to resources [81] which were provided.

Applying adult learning principles [38], clear objectives, relevant topics, a strong connection to learner’s real-life knowledge and experience, and maximising opportunities for interactivity were applied [83]. Whilst there were core learning objectives within the syllabus that needed to be met in order to provide evidence-based care and management for people living with CHB, there was also flexibility for our Aboriginal and Torres Strait Islander researchers and participants to shape course content. Consideration of various levels of English-language, literacy, health literacy and past formal learning experiences were all considered [51, 52]. Where language or written text was used, it was in plain language and avoided intangible concepts and idiom [78, 84]. The teach-back method was also employed. This involves clinicians clarifying with patients the information given to assess whether intended messages were appropriately being conveyed, to identify misunderstanding and initiate clarifications [58]. It has been shown to be effective in increasing understanding and retention of health information in a cross-cultural setting [58, 85] and in improving knowledge for people living with CHB [58]. We used this tool to assess our teaching and to introduce the concept and explain its utility, so participants could then also consider using the teach-back method in their professional roles with patients.

Creating culturally welcoming spaces has been identified as an enabler to cultural safety in Aboriginal and Torres Strait Islander health and education settings [86]. Choosing an appropriate venue, offering food and drink, and allowing appropriate time for breaks have also been influential factors in training success [79]. We consulted with local staff about food preferences as food provides comfort and a sense of emotional as well as nutritional sustenance [21].

### Implementation, sustainability, and transferability

Although we are supportive of providing training opportunities in community, with 76 remote communities in the NT [87], it is not financially viable, environmentally sustainable nor an effective way to deliver our program. Another consideration is the large emotional demands the Aboriginal heath workforce experience in their roles [88], their commitment to their communities often extends beyond standard working hours, with no marked separation between their ‘personal life’ and ‘professional life’ [1]. This responsibility risks exhaustion if not adequately supported and recognised [89]. Our findings indicated a preference for training out of community to allow space to learn without being disturbed by the pressures inherent to their roles. Our endorsed courses are being delivered in regional centres to allow greater access and equity to training opportunities, and in recognition of the risks associated with Aboriginal heath workforce’s responsibilities in community, and may be a strategy to reduce burnout, enhancing sustainability and retention of the workforce [88]. We describe the evaluation of the endorsed courses elsewhere [63].

Throughout our study we have strengthened and developed strong, trusted, and respectful relationships with participants and communities. We were able to harness these trusted networks at the beginning of the COVID-19 pandemic to provide information and used this model of education to engage, co-design, deliver and evaluate COVID-19 education for the Aboriginal health workforce, confirming the adaptability of the course co-design. This “Managing hepatitis B” course has received positive national attention and the syllabus has been shared with Aboriginal Community Controlled organisations in other states of Australia. Work is underway to extend its application and accessibility for greater reach. The authors encourage wide use and adaptation to local Aboriginal and Torres Strait Islander culture and context, ensuring Aboriginal and Torres Strait Islander colleagues participate in review and delivery of content.

## Conclusions

The Aboriginal health workforce are vital for providing accessible, equitable and culturally safe health care to their communities. To improve overall health equity for Aboriginal and Torres Strait Islander Peoples a structural, systemic process change needs to occur within health systems, including increasing educational and professional development opportunities for the Aboriginal health workforce. Education courses should be co-designed with critical self-reflection, critiquing power imbalances, biases, and privilege. Fundamentally, culturally safe education should be based on reciprocity, empowerment, and respect. To our knowledge this is the first study to describe a transferable framework for the development of a culturally safe training model for the Aboriginal health workforce.

### Supplementary Information

**Additional file 1.**

**Additional file 2.**

## Data Availability

Ethical and privacy considerations restrict public access to the data collected and analysed in this study. Data may be available for reasonable requests through to the Hep B PAST steering committee, email: Hepbpast@menzies.edu.au. Course materials and guidelines of use are available on request through the corresponding author.

## Abbreviations

AHP: Aboriginal Health Practitioner
ACW: Aboriginal Community Worker
CHB: Chronic hepatitis B
COVID-19: SARS-CoV-2
HBV: Hepatitis B virus
NATSIHWA: National Aboriginal and Torres Strait Islander Health Workers Association
NAATSIHWP: National Association of Aboriginal and Torres Strait Islander Health Workers and Practitioners
NHMRC: National Health and Medical Research Council
NT: Northern Territory
PAR: Participatory Action Research
