# Supplementary material for: “The most culturally safe training I’ve ever had”: the co-design of a culturally safe Managing hepatitis B training course with and for the Aboriginal health workforce of the Northern Territory of Australia
Source: BMC Health Serv Res. 2023 Aug 31;23:935. doi: 10.1186/s12913-023-09902-w (PMC10472722; doi:10.1186/s12913-023-09902-w)
Supplement: Supplementary file 1 — Additional file 1. [file 12913_2023_9902_MOESM1_ESM.pdf]

Additional file 1: Process to develop and assess evaluation tools and tools selected for use in future courses.

Method	Number of participants	Tool 1 (Appropriate?)	Tool 2 (Appropriate?)	Tool 3 (Appropriate?)	Tool 4 (Appropriate?)	Tool 5 (Appropriate?)	Tool 6 (Appropriate?)	Decision/preference
**Focus group (1)**<br>First meeting	4	No	Yes	Yes	Not yet developed	Not yet developed	Not yet developed	Trial tool 2 & 3<br>Do not use tool 1
**In class (pilot 1)**<br>Small group discussion & observation	7	Not used	No	Yes	Yes	Not yet developed	Not yet developed	Continue to use tool 3.<br>Trial tool 2 again to obtain further information on acceptability.<br>Tool 4 developed
**Focus group (1)**<br>Second meeting	4	Not used	Maybe	Yes	Yes	Yes	Not yet developed	Tool 3 acceptable. Tool 2 needs another trial. Tool 4 acceptable to help shape course design and clarify. Tool 5 developed.
**In class (pilot 2)**<br>Small group discussion & observation	10	Not used	No	Yes	Yes	Yes	Not yet developed	Tools 3 and 5 preferred and acceptable. Tool 4 acceptable, complementary to teach-back method. Tool 2 mixed review, some confusion using the scale
**Focus group (1)**<br>Third meeting	6	Not used	No	Yes	Yes	Yes	Yes	Decided to no longer use Tool 2.<br>Tool 3, 4, 5 acceptable.<br>Tool 5 modified to make questions clearer.<br>Discussed adding semi-structured interviews for future course, questions designed.
**Focus group (2)**<br>Indigenous reference group	8	Not used	No	Yes	Yes	Yes	Yes	Tool 5 preferred. Tool 3, 6 acceptable. Decision, use tools 3, 5 and 6 to assess course outcomes & tool 4 as a flexible facilitation tool, with ability to assess individual's knowledge through observing case studies or for groups.

Legend:

Tool 1: Generic smiley face evaluation tool.

Tool 2: Scale of strongly agree to strongly disagree, with questions requiring written responses, assessing understanding and acceptability.

Tool 3: Butchers paper tool with three questions; What did you like about the course? What could be improved about the course? What was one thing that you learnt?

Tool 4: Knowledge assessment tool: observation of case studies

Tool 5: Questionnaire developed with the focus group, measuring knowledge, attitude, and practice - with yes, no, don't know scale options.

Tool 6: Semi-structured interview questions

The process to develop and assess evaluation tools involved seven sessions, in four locations. Six different evaluation tools were discussed, observed in use, and evaluated. Results from focus group discussions, in-class observations, and voting, showed that tools 3 and 5 [see below] were acceptable. These tools were selected for use in future courses. The semi-structured interview questions were developed after pilot 2 as the need to gather richer qualitative data was identified. This tool, 6, [see below], along with tool 3 and 5, were trialled in the first finalised course in Darwin, 2019. Tool 4, the "knowledge assessment tool: observation of case studies" (see below) was acceptable to facilitators for assessing knowledge and readiness to be active members of the care team for people living with CHB. Where consent was given by the participant, their knowledge assessment tool was sent to their manager to highlight areas of competency and strength and areas where additional support or mentoring may be required.

Hep B PAST

Tool 4: Knowledge Assessment Tool: Observations of Case Studies

Name of participant_____________________________

Name of facilitator __________________________

**Topic**	**Examples (circle)**	**Discussed (circle)**	**Correct (circle)**	**Score (circle)**
What is Hep B	• Virus<br>• Silent for long time<br>• Affects the liver.<br>• Changes over time	Y / N	Y / N	1 – needs improvement<br>2 – some knowledge<br>3 - good knowledge
What does the liver do?	• Cleans blood<br>• Help fight infection.<br>• Helps digestion.<br>• Helps clot blood	(at least 1 point)<br><br>Y/ N	Y/ N	1 – needs improvement<br>2 – some knowledge<br>3 - good knowledge
Where is liver?	• Pointing is ok<br>• Right side	Y/ N	Y / N	1 – needs improvement<br>2 – some knowledge<br>3 - good knowledge
How is it passed on	**Main**<br>• Early childhood<br>• Maternal to child transmission<br>**Other**<br>• Blood<br>• Sport<br>• Sex<br>• Ceremony / razors/ toothbrushes	Y / N	Y / N	1 – needs improvement<br>2 – some knowledge<br>3 - good knowledge
How common	• 1 in 10 in NT<br>• All over world	Y / N	Y / N	1 – needs improvement<br>2 – some knowledge<br>3 - good knowledge
How to stop getting it	• Vaccinations<br>• Safer sex<br>• Know your status (get blood test)	Y / N	Y / N	1 – needs improvement<br>2 – some knowledge<br>3 - good knowledge
If you have Hep B<br>Non treatment phase	• Clinic every 6 months<br>• Healthy living	Y/ N	Y / N	1 – needs improvement<br>2 – some knowledge<br>3 - good knowledge
If you have Hep B<br>Needs treatment	• Clinic every 6 months<br>• Healthy living<br>• Medicines daily	Y/ N	Y / N	1 – needs improvement<br>2 – some knowledge<br>3 - good knowledge
Health Promotion	• Stop drinking (Drink less or preferably, none)<br>• Eat healthy and maintain a healthy weight.<br>• Reduced smoking.<br>• Exercise regularly	Y/N	Y/N	1 – needs improvement<br>2 – some knowledge<br>3 - good knowledge

Copyright © 2021 Hep B PAST, Menzies School of Health Research. All rights reserved

Facilitator debriefing____

Participant name____

____
____
____
____
____
____
____
____
____
____

Overall impression

1 – needs improvement

2 – some knowledge

3 - good knowledge

Copyright © 2021 Hep B PAST, Menzies School of Health Research. All rights reserved

Tool 5: Pre-training questionnaire for participants

Managing Hepatitis B for Aboriginal Health Workforce

What is this questionnaire?

- Before we begin the course, we would like to ask you to participate in this pre-learning questionnaire. The purpose of these questions is to collect information from learners about their knowledge, attitudes and skills prior to participating in the course. We will ask you the same questions at the end of the course.
- This will help us assess if we are teaching well or need to change the course.
- The questionnaire will be analysed as a whole group, not individually. The findings may be used as a report or presentation about the course evaluation, but individual answers will be confidential.
- If you need any of the questions explained or clarified please ask the presenter or facilitator.
- If you would like a copy of the final report of the evaluation, please contact Kelly Hosking, Top End Health Service, kelly.hosking@nt.gov.au Ph: 0472817647

Do you give consent for your questionnaire being used for research purposes?

Yes ☐ No ☐

Copyright © 2021 Hep B PAST, Menzies School of Health Research. All rights reserved

General Information

1. Your name or initials:

2. Please circle:

   a. Female   b. Male   c. Other

3. What is your job?
   a. Aboriginal Health Practitioner
   b. Aboriginal Community Worker
   c. Alcohol and other drugs
   d. Families as first teachers (FaFT)
   e. Other (please specify)_______________________

4. What is your age group?
   a. 19 years or younger
   b. 20-24 years old
   c. 25-29 years old
   d. 30-34 years old
   e. 35-39 years old
   f. 40-44 years old
   g. 45-49 years old
   h. 50-54 years old
   i. 55 or older

Copyright © 2021 Hep B PAST, Menzies School of Health Research. All rights reserved

Knowledge

Please put a cross (X) in which answer you think is correct for each question 1-11, if you don't know that's ok, please put a cross (X) in "I don't know"

	Yes	No	I don't know
1. Is hepatitis B common in Aboriginal people in the NT?			
2. Is hepatitis B a problem for the liver?			
3. Can you get hepatitis B virus from drinking grog?			
4. The main way people get hepatitis B is in early childhood?			
5. Can hepatitis B lead to liver cancer?			

Attitude

	Yes	No	I don't know
6. Is it a person's own fault if they have hepatitis B?			
7. Is it safe for a person with hepatitis B to share meals with their family?			

Perception about skills (professional role)

	Yes	No	I don't know
8. Do you feel confident talking to clients, family, or community about hepatitis B?			
9. Do you know how to use the Hep B Story app?			
10. Do you know what you'd tell someone about keeping their liver healthy?			
11. Do you how to explain the main ways you can get hepatitis B?			

Copyright © 2021 Hep B PAST, Menzies School of Health Research. All rights reserved

Tool 5: Post-training questionnaire for participants

Managing Hepatitis B for Aboriginal Health Workforce

What is this questionnaire?

- Now you have finished the course we would like to ask you to participate in this post-learning questionnaire. The purpose of these questions is to collect information from learners about their knowledge, attitudes, and skills after participating in the course.
- This will help us assess if we are teaching well or need to change the course.
- The questionnaire will be analysed as a whole group, not individually. The findings may be used as a report or presentation about the course evaluation, but individual answers will be confidential.
- If you need any of the questions explained or clarified, please ask the presenter or facilitator
- If you would like a copy of the final report of the evaluation, please contact Kelly Hosking, Top End Health Service, kelly.hosking@nt.gov.au Ph: 0472817647

Do you give consent for your questionnaire being used for research purposes?

Yes ☐ No ☐

Copyright © 2021 Hep B PAST, Menzies School of Health Research. All rights reserved

General Information

5. Your name or initials:

6. Please circle:

   a. Female b. Male c. Other

7. What is your job?
   a. Aboriginal Health Practitioner
   b. Aboriginal Community Worker
   c. Alcohol and other drugs
   d. Families as first teachers (FaFT)
   e. Other (please specify) ________________________

8. What is your age group?
   a. 19 years or younger
   b. 20-24 years old
   c. 25-29 years old
   d. 30-34 years old
   e. 35-39 years old
   f. 40-44 years old
   g. 45-49 years old
   h. 50-54 years old
   i. 55 or older

Copyright © 2021 Hep B PAST, Menzies School of Health Research. All rights reserved

Knowledge

Please put a cross (X) in which answer you think is correct for each question 1-11, if you don't know that's ok, please put a cross (X) in "I don't know"

	Yes	No	I don't know
1. Is hepatitis B common in Aboriginal people in the NT?			
2. Is hepatitis B a problem for the liver?			
3. Can you get hepatitis B virus from drinking grog?			
4. The main way people get hepatitis B is in early childhood?			
5. Can hepatitis B lead to liver cancer?			

Attitude

	Yes	No	I don't know
6. Is it a person's own fault if they have hepatitis B?			
7. Is it safe for a person with hepatitis B to share meals with their family?			

Perception about skills (professional role)

	Yes	No	I don't know
8. Do you feel confident talking to clients, family, or community about hepatitis B?			
9. Do you know how to use the Hep B Story app?			
10. Do you know what you'd tell someone about keeping their liver healthy?			
11. Do you know how to explain the main ways you can get hepatitis B?			

Copyright © 2021 Hep B PAST, Menzies School of Health Research. All rights reserved

Hep B PAST

Participant feedback to the Hepatitis B Course

Please put a cross (X) in the most appropriate answer. If you don't know please put a cross (X) in "I don't know."

	Yes	No	I don't know
1. The course was interesting			
2. The case studies were helpful for my learning			
3. The learning resources (games, handouts) were useful			
4. The presenters had a good understanding of hepatitis B			
5. The facilitators were helpful to my learning			

What did you like about the course?

What did you NOT like about the course?

What was something you learnt about hepatitis B?

Do you have any other suggestions / feedback?

Copyright © 2021 Hep B PAST, Menzies School of Health Research. All rights reserved

Tool 6: Semi Structure Interview questions
Evaluation of course: Aboriginal Workforce Hepatitis B Course

Please record and have transcribed.

Can pair up AHP/ACWs if you think it would benefit someone to have some translating or discussion in-language.

1. What did you think of the Hep B course? (Prompt if necessary: interesting, clear, met expectations)
2. What was the most valuable thing you learnt?
3. What was the hardest thing about the course?
4. What was something you learnt?
5. What did you think about the case studies?
6. What could be improved about the course?
7. What did you think about the length of the course? (prompt: too long, too short)
8. Is there anything you wanted to spend more time on?
9. What messages do you think you could share or teach with clients or family?
10. What could help you use the information you have learnt in the community? (Prompt: support from manager, booklet, AHP coordinators)

	**Yes**	**No**	**I don't know**
6. The learning resources (videos, handouts) were useful			
7. The presenter had a good understanding of *hepatitis B*			
8. The facilitators were helpful to my learning			

Copyright © 2021 Hep B PAST, Menzies School of Health Research. All rights reserved
