# Supplementary material for: “The most culturally safe training I’ve ever had”: the co-design of a culturally safe Managing hepatitis B training course with and for the Aboriginal health workforce of the Northern Territory of Australia
Source: BMC Health Serv Res. 2023 Aug 31;23:935. doi: 10.1186/s12913-023-09902-w (PMC10472722; doi:10.1186/s12913-023-09902-w)
Supplement: Supplementary file 2 — Additional file 2. [file 12913_2023_9902_MOESM2_ESM.pdf]

Hep B PAST Partnership Members and Individuals

Partner Organisations

Menzies School of Health Research
Northern Territory Government, NT Health
ASHM
Katherine West Health Board Aboriginal Corporation
Miwatj Health Aboriginal Corporation
NT AIDS and Hepatitis Council
Mala'la Health Service Aboriginal Corporation
Marthakal Homelands Health Service
Central Australian Aboriginal Congress

Associate Partner Organisations

WHO Collaborating Centre for Viral Hepatitis, VIDRL, Doherty Institute
VIDRL
Hepatitis Australia

Investigators and Contributors

Adrian Miller	Letisha Parker
Amanda Dhagapan	Levinia Crooks^
Anna Deng	Libby Coombes
Anna Ralph	Linda Bunn
Anngie Everitt	Lorraine Johns
Ashleigh Qama	Lou Sanderson
Barbara De Graaff	Lucie Perriseel
Belinda Greenwood-Smith	Manoji Gunathilake
Benjamin Cowie	Marco Briceno
Brianna Summers	Margaret Littlejohn
Carrie Fowler	Maria Scarlett
Catherine Blacker	Marilou Capati
Catherine Gargan	Matthew Maddison
Catherine Marshall	Melita McKinnon
Catherine Stoddart	Mikaela Mobsby
Charles Pain	Molly Shorthouse
Cheryl Ross	Monica Ostigh
Christine Connors	Nicole Allard
David Boettiger	Nicole Romero

David McGuinness	Paula Binks
David Reeve	Penny Ramsey
Deb Gent	Peter Nihill
Dee Hampton	Phillip Merrdi Wilson
Eddie Mulholland	Phoebe Schroder
Ella Meumann	Prashanti Manchikanti
Emily Vintour-Cesar	Rebecca Katiforis
Emma Childs	Richard Sullivan
Genevieve Dally	Robert Batey
Geoffrey Stewart	Roisin Steward
George Garambaka Gurruwiwi	Roslyn Dhurrkay
Hayden Jose	Sami Stewart
Helen Goodwin	Sandra Nelson
Hillary Bloomfield	Sarah Mariyalawuy Bukulatjpi
Hugh Heggie	Sean Heffernan
Isabelle Purcell	Sean Taylor
Jaclyn Tate-Baker	Shiraline Wurrawilya
Jane Davies	Sinon Cooney
Jessica Michaels	Sonja Hill
John Boffa	Stephen Locarnini
Joshua Davis	Steven Skov
Jyoti Jadeja	Steven Tong
Karen Fuller	Su Govindasamy
Katherine McNamara	Sudharsan Venkatesan
Katie McGuire	Tammy-Allyn Fernandes
Keith Forrest	Tanya Plavins
Kelly Banz	Teresa De Santis
Kelly Hosking	Teresa Ngurruwuthun
Kelly-Anne Stuart-Carter	Tiana Alley
Kerrie Jordan	Timothy Nabegeyo
Khim Tan	Vanessa Towell
Leanne O'Connor	Vicki Krause
Wendy Page	Megan Howitt
